# 4-[(2′-Cyano­biphenyl-4-yl)methyl]­morpholin-4-ium nitrate

**DOI:** 10.1107/S1600536810027443

**Published:** 2010-07-17

**Authors:** Weiwei SiMa

**Affiliations:** a Ordered Matter Science Research Center, Southeast University, Nanjing 210096, People’s Republic of China

## Abstract

The title ion pair, C_18_H_19_N_2_O^+^·NO_3_^−^, features an N—H⋯O hydrogen bond linking the cation to the anion. The morpholine portion adopts a chair conformation; the aromatic rings of the biphenyl­ene portion are twisted [torsion angles for the four atoms involving the ar­yl–aryl bond = 35.1 (2)–40.4 (2)°].

## Related literature

For the synthesis, see: Li *et al.* (2008 ▶); Zhang *et al.* (2009 ▶).
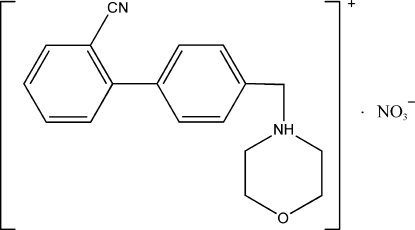


## Experimental

### 

#### Crystal data

C_18_H_19_N_2_O^+^·NO_3_^−^
*M* *_r_* = 341.36
Monoclinic, 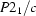

*a* = 12.670 (6) Å
*b* = 13.120 (5) Å
*c* = 10.865 (5) Å
β = 110.927 (8)°
*V* = 1687.0 (12) Å^3^
*Z* = 4
Mo *K*α radiation
μ = 0.10 mm^−1^
*T* = 293 K
0.20 × 0.20 × 0.20 mm

#### Data collection

Rigaku SCXmini diffractometer
Absorption correction: multi-scan (*CrystalClear*; Rigaku, 2005 ▶) *T*_min_ = 0.981, *T*_max_ = 0.981
18242 measured reflections
3852 independent reflections
2848 reflections with *I* > 2σ(*I*)
*R*_int_ = 0.052

#### Refinement

*R*[*F* ^2^ > 2σ(*F* ^2^)] = 0.062
*wR*(*F* ^2^) = 0.178
*S* = 1.17
3852 reflections
226 parameters
H-atom parameters constrained
Δρ_max_ = 0.24 e Å^−3^
Δρ_min_ = −0.24 e Å^−3^

### 

Data collection: *CrystalClear* (Rigaku, 2005 ▶); cell refinement: *CrystalClear*; data reduction: *CrystalClear*; program(s) used to solve structure: *SHELXS97* (Sheldrick, 2008 ▶); program(s) used to refine structure: *SHELXL97* (Sheldrick, 2008 ▶); molecular graphics: *SHELXTL* (Sheldrick, 2008 ▶); software used to prepare material for publication: *SHELXL97*.

## Supplementary Material

Crystal structure: contains datablocks I, global. DOI: 10.1107/S1600536810027443/ng2795sup1.cif

Structure factors: contains datablocks I. DOI: 10.1107/S1600536810027443/ng2795Isup2.hkl

Additional supplementary materials:  crystallographic information; 3D view; checkCIF report

## Figures and Tables

**Table 1:** Hydrogen-bond geometry (Å, °)

*D*—H⋯*A*	*D*—H	H⋯*A*	*D*⋯*A*	*D*—H⋯*A*
N2—H2*A*⋯O2	0.91	1.88	2.784 (2)	172

**Fig. 1.**
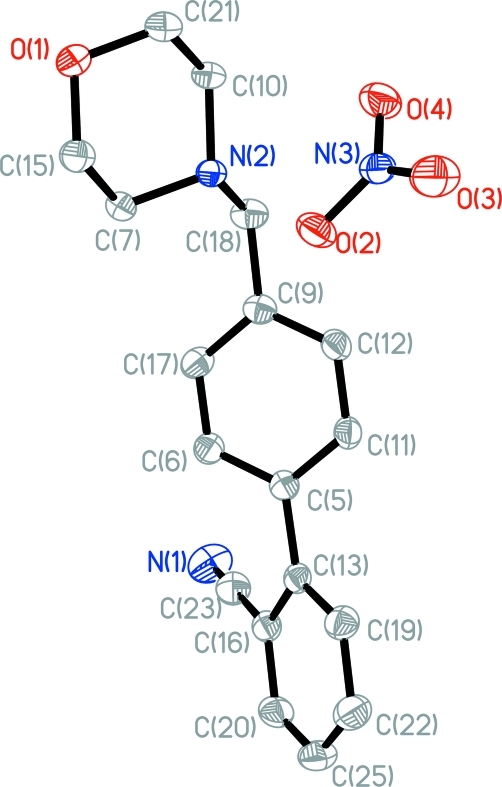
The molecular structure of the title compound, with the atomic numbering scheme. Displacement ellipsoids are drawn at the 30% probability level.

**Fig. 2.**
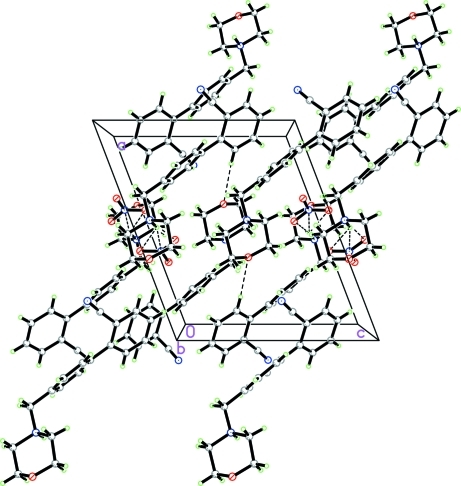
A view of the packing of the title compound, stacking along the b axis. Dashed lines indicate hydrogen bonds.

## supplementary crystallographic information

### Comment

As a continuation of our study of dielectric-ferroelectric materials, including organic ligands (Li *et al.*, 2008), metal-organic coordination compounds (Zhang *et al.*, 2009), organic-inorganic hybrids, we are interested in the dielectric properties (capacitance and dielectric loss measurements) of the title compound(I), unfortunately, there was no distinct anomaly observed from 93 K to 350 K. In thisarticle, the crystal structure of (I) has been presented.

The asymmetric unit of the title compound consists of one 4'-morpholinemethylbiphenyl-2-carbonitrile cation and one nitrate e-66-o2042-fig1). The intermolecular N—H···O, N—H···N hydrogen bonds link the cations and anions to chains along *b*e-66-o2042-fig2), and make great contribution to the stability of the structure.The title compound crystallizes in the monoclinic system, space groupP2_1_/c.

### Experimental

4'-morpholinemethylbiphenyl-2-carbonitrile (10 mmol)was dissolved in 10 ml e thanol, to which nitrate acid(10 mmol) was added dropwise under stirring, the reaction solution was stirred for a few minutes.water was added untill all suspended substrates disappeared. Colorless crystals suitable for X-ray analysis were formed after several days by slow evaporation of the solvent at room temperature.

### Refinement

Positional parameters of all the H atoms were calculated geometrically and were allowed to ride on the C, N atoms to which they are bonded, with C—H =0.93 to 0.97 Å, *U*_iso_(H) = 1.2 *U*_eq_(C), N—H = 0.91 Å, *U*_iso_(H)= 1.5 *U*_eq_(N).

### Crystal data

**Table:**

C_18_H_19_N_2_O^+^·NO_3_^−^	*F*(000) = 720
*M**_r_* = 341.36	*D*_x_ = 1.344 Mg m^−^^3^
Monoclinic, *P*2_1_/*c*	Mo *K*α radiation, λ = 0.71073 Å
Hall symbol: -P 2ybc	Cell parameters from 3727 reflections
*a* = 12.670 (6) Å	θ = 2.3–27.5°
*b* = 13.120 (5) Å	µ = 0.10 mm^−^^1^
*c* = 10.865 (5) Å	*T* = 293 K
β = 110.927 (8)°	Prism, colourless
*V* = 1687.0 (12) Å^3^	0.20 × 0.20 × 0.20 mm
*Z* = 4	

### Data collection

**Table:**

Rigaku SCXmini diffractometer	3852 independent reflections
Radiation source: fine-focus sealed tube	2848 reflections with *I* > 2σ(*I*)
graphite	*R*_int_ = 0.052
Detector resolution: 13.6612 pixels mm^-1^	θ_max_ = 27.5°, θ_min_ = 1.7°
ω scans	*h* = −16→16
Absorption correction: multi-scan (*CrystalClear*; Rigaku, 2005)	*k* = −17→17
*T*_min_ = 0.981, *T*_max_ = 0.981	*l* = −14→14
18242 measured reflections	

### Refinement

**Table:**

Refinement on *F*^2^	Primary atom site location: structure-invariant direct methods
Least-squares matrix: full	Secondary atom site location: difference Fourier map
*R*[*F*^2^ > 2σ(*F*^2^)] = 0.062	Hydrogen site location: inferred from neighbouring sites
*wR*(*F*^2^) = 0.178	H-atom parameters constrained
*S* = 1.17	*w* = 1/[σ^2^(*F*_o_^2^) + (0.0839*P*)^2^ + 0.0408*P*] where *P* = (*F*_o_^2^ + 2*F*_c_^2^)/3
3852 reflections	(Δ/σ)_max_ < 0.001
226 parameters	Δρ_max_ = 0.24 e Å^−^^3^
0 restraints	Δρ_min_ = −0.24 e Å^−^^3^

### Special details

**Table:**

Geometry. All e.s.d.'s (except the e.s.d. in the dihedral angle between two l.s. planes) are estimated using the full covariance matrix. The cell e.s.d.'s are taken into account individually in the estimation of e.s.d.'s in distances, angles and torsion angles; correlations between e.s.d.'s in cell parameters are only used when they are defined by crystal symmetry. An approximate (isotropic) treatment of cell e.s.d.'s is used for estimating e.s.d.'s involving l.s. planes.
Refinement. Refinement of *F*^2^ against ALL reflections. The weighted *R*-factor *wR* and goodness of fit *S* are based on *F*^2^, conventional *R*-factors *R* are based on *F*, with *F* set to zero for negative *F*^2^. The threshold expression of *F*^2^ > σ(*F*^2^) is used only for calculating *R*-factors(gt) *etc*. and is not relevant to the choice of reflections for refinement. *R*-factors based on *F*^2^ are statistically about twice as large as those based on *F*, and *R*- factors based on ALL data will be even larger.

### Fractional atomic coordinates and isotropic or equivalent isotropic displacement parameters (Å^2^)

**Table:**

	*x*	*y*	*z*	*U*_iso_*/*U*_eq_	
N2	0.45201 (13)	0.21560 (12)	0.92144 (15)	0.0332 (4)	
H2A	0.4862	0.2716	0.9032	0.040*	
O1	0.63116 (14)	0.06839 (12)	0.98832 (15)	0.0521 (5)	
C5	0.12498 (16)	0.35987 (15)	0.5246 (2)	0.0358 (5)	
C6	0.12785 (18)	0.25575 (16)	0.5517 (2)	0.0417 (5)	
H6A	0.0840	0.2110	0.4873	0.050*	
C7	0.45639 (18)	0.13253 (16)	0.8303 (2)	0.0392 (5)	
H7A	0.4217	0.1556	0.7399	0.047*	
H7B	0.4140	0.0742	0.8423	0.047*	
C9	0.26229 (17)	0.28322 (17)	0.7722 (2)	0.0393 (5)	
C10	0.51627 (19)	0.18427 (17)	1.06051 (19)	0.0430 (5)	
H10A	0.4767	0.1291	1.0849	0.052*	
H10B	0.5206	0.2412	1.1190	0.052*	
C11	0.19283 (18)	0.42418 (16)	0.6230 (2)	0.0419 (5)	
H11A	0.1930	0.4937	0.6065	0.050*	
C12	0.26004 (18)	0.38675 (17)	0.7450 (2)	0.0441 (5)	
H12A	0.3041	0.4314	0.8093	0.053*	
C13	0.05740 (17)	0.40207 (15)	0.3931 (2)	0.0371 (5)	
C15	0.5770 (2)	0.10142 (19)	0.8560 (2)	0.0485 (6)	
H15A	0.5782	0.0467	0.7964	0.058*	
H15B	0.6181	0.1588	0.8389	0.058*	
C16	−0.05099 (18)	0.36520 (16)	0.3153 (2)	0.0416 (5)	
C17	0.19541 (18)	0.21840 (17)	0.6737 (2)	0.0447 (5)	
H17A	0.1961	0.1488	0.6901	0.054*	
C18	0.33288 (18)	0.24421 (19)	0.9069 (2)	0.0459 (6)	
H18A	0.2960	0.1849	0.9265	0.055*	
H18B	0.3359	0.2962	0.9716	0.055*	
C19	0.1012 (2)	0.47979 (17)	0.3390 (2)	0.0482 (6)	
H19A	0.1713	0.5072	0.3883	0.058*	
C20	−0.1077 (2)	0.40316 (19)	0.1893 (2)	0.0533 (6)	
H20A	−0.1783	0.3774	0.1390	0.064*	
C21	0.63421 (19)	0.14995 (18)	1.0763 (2)	0.0494 (6)	
H21A	0.6758	0.2068	1.0588	0.059*	
H21B	0.6734	0.1278	1.1663	0.059*	
C22	0.0438 (2)	0.5178 (2)	0.2140 (3)	0.0586 (7)	
H22A	0.0755	0.5701	0.1807	0.070*	
C23	−0.11159 (19)	0.29425 (19)	0.3668 (2)	0.0513 (6)	
N1	−0.1659 (2)	0.2409 (2)	0.4032 (3)	0.0747 (7)	
C25	−0.0596 (2)	0.4786 (2)	0.1392 (3)	0.0606 (7)	
H25A	−0.0972	0.5031	0.0545	0.073*	
O2	0.54586 (15)	0.38211 (13)	0.83891 (15)	0.0550 (5)	
N3	0.60177 (17)	0.43391 (14)	0.93873 (18)	0.0465 (5)	
O4	0.60072 (17)	0.40818 (14)	1.04806 (16)	0.0646 (5)	
O3	0.65691 (17)	0.50803 (15)	0.92616 (18)	0.0771 (6)	

### Atomic displacement parameters (Å^2^)

**Table:**

	*U*^11^	*U*^22^	*U*^33^	*U*^12^	*U*^13^	*U*^23^
N2	0.0332 (9)	0.0350 (9)	0.0292 (9)	−0.0012 (7)	0.0086 (7)	0.0015 (7)
O1	0.0519 (10)	0.0499 (10)	0.0448 (9)	0.0160 (7)	0.0054 (8)	0.0012 (7)
C5	0.0265 (9)	0.0401 (11)	0.0396 (11)	0.0025 (8)	0.0102 (8)	−0.0027 (9)
C6	0.0344 (11)	0.0383 (12)	0.0457 (12)	−0.0037 (9)	0.0063 (9)	−0.0019 (9)
C7	0.0430 (12)	0.0375 (11)	0.0322 (11)	−0.0007 (9)	0.0075 (9)	−0.0028 (8)
C9	0.0290 (10)	0.0500 (12)	0.0385 (12)	0.0021 (9)	0.0115 (9)	0.0009 (9)
C10	0.0486 (13)	0.0479 (13)	0.0275 (10)	0.0041 (10)	0.0073 (9)	0.0029 (9)
C11	0.0402 (11)	0.0350 (11)	0.0454 (12)	0.0045 (9)	0.0089 (10)	−0.0062 (9)
C12	0.0394 (11)	0.0441 (12)	0.0418 (12)	0.0035 (9)	0.0059 (10)	−0.0089 (9)
C13	0.0327 (10)	0.0347 (11)	0.0406 (11)	0.0066 (8)	0.0092 (9)	−0.0026 (9)
C15	0.0489 (13)	0.0535 (14)	0.0397 (12)	0.0093 (11)	0.0116 (11)	−0.0016 (10)
C16	0.0348 (11)	0.0423 (12)	0.0436 (12)	0.0057 (9)	0.0089 (10)	−0.0037 (9)
C17	0.0367 (11)	0.0400 (12)	0.0508 (13)	−0.0032 (9)	0.0077 (10)	0.0064 (10)
C18	0.0361 (11)	0.0621 (15)	0.0405 (12)	0.0040 (10)	0.0149 (10)	0.0049 (10)
C19	0.0422 (12)	0.0469 (13)	0.0503 (14)	0.0010 (10)	0.0102 (11)	0.0017 (10)
C20	0.0441 (13)	0.0616 (16)	0.0428 (13)	0.0069 (11)	0.0015 (11)	−0.0046 (11)
C21	0.0446 (13)	0.0540 (14)	0.0388 (12)	0.0050 (10)	0.0018 (10)	−0.0002 (10)
C22	0.0632 (16)	0.0534 (15)	0.0562 (15)	0.0029 (12)	0.0178 (13)	0.0099 (12)
C23	0.0306 (11)	0.0584 (15)	0.0557 (15)	0.0016 (10)	0.0043 (11)	−0.0023 (12)
N1	0.0444 (13)	0.0834 (18)	0.0884 (18)	−0.0072 (12)	0.0139 (13)	0.0157 (14)
C25	0.0644 (17)	0.0659 (17)	0.0423 (13)	0.0150 (13)	0.0075 (12)	0.0088 (12)
O2	0.0617 (11)	0.0570 (10)	0.0381 (9)	−0.0137 (8)	0.0075 (8)	−0.0069 (7)
N3	0.0480 (11)	0.0412 (11)	0.0392 (11)	−0.0038 (8)	0.0022 (9)	0.0047 (8)
O4	0.0876 (14)	0.0602 (11)	0.0383 (9)	−0.0121 (9)	0.0129 (9)	0.0019 (8)
O3	0.0796 (14)	0.0641 (12)	0.0676 (13)	−0.0319 (10)	0.0018 (11)	0.0168 (10)

### Geometric parameters (Å, °)

**Table:**

N2—C7	1.487 (3)	C13—C19	1.388 (3)
N2—C10	1.495 (3)	C13—C16	1.416 (3)
N2—C18	1.508 (3)	C15—H15A	0.9700
N2—H2A	0.9100	C15—H15B	0.9700
O1—C15	1.422 (3)	C16—C20	1.391 (3)
O1—C21	1.426 (3)	C16—C23	1.441 (3)
C5—C11	1.392 (3)	C17—H17A	0.9300
C5—C6	1.395 (3)	C18—H18A	0.9700
C5—C13	1.485 (3)	C18—H18B	0.9700
C6—C17	1.386 (3)	C19—C22	1.384 (3)
C6—H6A	0.9300	C19—H19A	0.9300
C7—C15	1.508 (3)	C20—C25	1.372 (4)
C7—H7A	0.9700	C20—H20A	0.9300
C7—H7B	0.9700	C21—H21A	0.9700
C9—C12	1.388 (3)	C21—H21B	0.9700
C9—C17	1.392 (3)	C22—C25	1.372 (4)
C9—C18	1.506 (3)	C22—H22A	0.9300
C10—C21	1.511 (3)	C23—N1	1.146 (3)
C10—H10A	0.9700	C25—H25A	0.9300
C10—H10B	0.9700	O2—N3	1.261 (2)
C11—C12	1.384 (3)	N3—O3	1.233 (2)
C11—H11A	0.9300	N3—O4	1.240 (2)
C12—H12A	0.9300		
			
C7—N2—C10	109.59 (16)	C7—C15—H15A	109.4
C7—N2—C18	112.76 (16)	O1—C15—H15B	109.4
C10—N2—C18	109.72 (16)	C7—C15—H15B	109.4
C7—N2—H2A	108.2	H15A—C15—H15B	108.0
C10—N2—H2A	108.2	C20—C16—C13	121.0 (2)
C18—N2—H2A	108.2	C20—C16—C23	117.0 (2)
C15—O1—C21	109.68 (17)	C13—C16—C23	121.7 (2)
C11—C5—C6	117.98 (19)	C6—C17—C9	121.2 (2)
C11—C5—C13	119.90 (19)	C6—C17—H17A	119.4
C6—C5—C13	122.03 (19)	C9—C17—H17A	119.4
C17—C6—C5	120.6 (2)	C9—C18—N2	114.26 (17)
C17—C6—H6A	119.7	C9—C18—H18A	108.7
C5—C6—H6A	119.7	N2—C18—H18A	108.7
N2—C7—C15	110.57 (17)	C9—C18—H18B	108.7
N2—C7—H7A	109.5	N2—C18—H18B	108.7
C15—C7—H7A	109.5	H18A—C18—H18B	107.6
N2—C7—H7B	109.5	C22—C19—C13	122.1 (2)
C15—C7—H7B	109.5	C22—C19—H19A	119.0
H7A—C7—H7B	108.1	C13—C19—H19A	119.0
C12—C9—C17	118.2 (2)	C25—C20—C16	120.1 (2)
C12—C9—C18	120.0 (2)	C25—C20—H20A	119.9
C17—C9—C18	121.8 (2)	C16—C20—H20A	119.9
N2—C10—C21	110.85 (18)	O1—C21—C10	111.03 (18)
N2—C10—H10A	109.5	O1—C21—H21A	109.4
C21—C10—H10A	109.5	C10—C21—H21A	109.4
N2—C10—H10B	109.5	O1—C21—H21B	109.4
C21—C10—H10B	109.5	C10—C21—H21B	109.4
H10A—C10—H10B	108.1	H21A—C21—H21B	108.0
C12—C11—C5	121.4 (2)	C25—C22—C19	120.1 (3)
C12—C11—H11A	119.3	C25—C22—H22A	120.0
C5—C11—H11A	119.3	C19—C22—H22A	120.0
C11—C12—C9	120.7 (2)	N1—C23—C16	175.7 (3)
C11—C12—H12A	119.7	C20—C25—C22	120.1 (2)
C9—C12—H12A	119.7	C20—C25—H25A	120.0
C19—C13—C16	116.55 (19)	C22—C25—H25A	120.0
C19—C13—C5	120.05 (19)	O3—N3—O4	121.4 (2)
C16—C13—C5	123.37 (19)	O3—N3—O2	119.7 (2)
O1—C15—C7	111.16 (18)	O4—N3—O2	118.84 (19)
O1—C15—H15A	109.4		
			
C11—C5—C6—C17	0.8 (3)	C5—C13—C16—C23	9.9 (3)
C13—C5—C6—C17	177.3 (2)	C5—C6—C17—C9	−0.2 (3)
C10—N2—C7—C15	−53.1 (2)	C12—C9—C17—C6	−0.3 (3)
C18—N2—C7—C15	−175.65 (17)	C18—C9—C17—C6	177.73 (19)
C7—N2—C10—C21	52.7 (2)	C12—C9—C18—N2	−90.3 (3)
C18—N2—C10—C21	177.06 (18)	C17—C9—C18—N2	91.7 (3)
C6—C5—C11—C12	−0.9 (3)	C7—N2—C18—C9	−61.1 (2)
C13—C5—C11—C12	−177.56 (19)	C10—N2—C18—C9	176.48 (18)
C5—C11—C12—C9	0.5 (3)	C16—C13—C19—C22	−1.7 (3)
C17—C9—C12—C11	0.1 (3)	C5—C13—C19—C22	176.6 (2)
C18—C9—C12—C11	−177.9 (2)	C13—C16—C20—C25	−1.0 (3)
C11—C5—C13—C19	36.9 (3)	C23—C16—C20—C25	173.4 (2)
C6—C5—C13—C19	−139.6 (2)	C15—O1—C21—C10	60.9 (2)
C11—C5—C13—C16	−144.9 (2)	N2—C10—C21—O1	−57.1 (2)
C6—C5—C13—C16	38.6 (3)	C13—C19—C22—C25	−0.1 (4)
C21—O1—C15—C7	−61.6 (2)	C20—C16—C23—N1	−30 (4)
N2—C7—C15—O1	58.4 (2)	C13—C16—C23—N1	144 (4)
C19—C13—C16—C20	2.3 (3)	C16—C20—C25—C22	−1.0 (4)
C5—C13—C16—C20	−176.0 (2)	C19—C22—C25—C20	1.5 (4)
C19—C13—C16—C23	−171.9 (2)		

### Hydrogen-bond geometry (Å, °)

**Table:**

*D*—H···*A*	*D*—H	H···*A*	*D*···*A*	*D*—H···*A*
N2—H2A···O2	0.91	1.88	2.784 (2)	172
N2—H2A···O4	0.91	2.48	3.158 (3)	131
N2—H2A···N3	0.91	2.53	3.404 (3)	160
